# Influence of depth and translucency on the color matching of single-shade resin composites: An in vitro study

**DOI:** 10.1590/0103-644020256074

**Published:** 2025-04-07

**Authors:** Paula Fernandes-e-Silva, Marcieli Dias Furtado, Adriana Fernandes da Silva, Evandro Piva, Noéli Boscato, Wellington Luiz de Oliveira da Rosa

**Affiliations:** 1 College of Dentistry, Federal University of Pelotas, Pelotas, RS, Brazil

**Keywords:** blending effect, chameleon effect, structural color, monochromatic resin composite

## Abstract

To evaluate whether the depth and translucency of the restoration influence the color matching of single-color resin composites, cylindrical cavities (2-and-4 mm depths) were prepared in acrylic teeth 62 shade (n=10). Restorations were performed with single-shade resin composites (OC: Omnichroma, Tokuyama; VU: Vittra APS Unique, FGM) and a control multi-shade (EO: Estelite Omega, Tokuyama). The color matching was measured in instrumental analysis (CIEDE2000 color difference formula (ΔE_00_)) and visual analysis (0 to 4 scale). The translucency of discs (2-and-4mm thickness) was obtained by color difference (ΔE_00_) between black and white background. Two-way ANOVA followed by the post-hoc Tukey's test was used to evaluate instrumental analysis, and Kruskal-Wallis followed by the post-hoc Tukey's test to evaluate color matching with a significance level of 5%. Restoration depths affected the ΔE_00_ values of OC and VU (p<0.05) (higher ΔE_00_ values for 4-mm depth and higher for OC and VU (p< 0.05)). The restoration depths did not influence the color matching in the visual analysis. VU and OC showed higher translucency scores than EO. At 2 mm, OC showed higher translucency values and there was no difference between OC and VU at 4 mm (p <0.05). The color matching of OC and VU was influenced by depth (instrumental analysis). While the OC had a similar color matching to EO at both depths, VU had the best color matching in both depths in the visual analysis. Single-shade materials had greater translucency than EO at different depths, and the translucency decreased with increasing depth.

**Figure:**
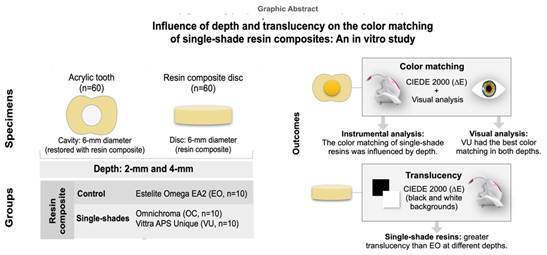

## Introduction

Direct resin composite restorations are the most often material satisfactorily used in dentistry in several clinical situations ^1^. For esthetically satisfactory restorations, the human eye must not be able to detect color differences between resin composite and the natural tooth substrate. However, this mentioned matching effect is linked to significant challenges related to the complexity of shade selection since teeth have a polychromatic nature and the resin composite has a limited number of shades ^2^. Thus, shade selection may require a longer chair time and an experienced operator ^3^. To reduce the material limitations and the sensitivity of the technique, single-shade resin composites have proposed a universal color that may match any tooth color ^1^.

Single-shade resin composites seem to have a blend effect property, also called the “chameleon effect, color adjustment potential, or color shifting” ^3^. Thus, the material takes on the color of surrounding tooth structures, which is possible through light reflection effects ^4^. When light reflects on the surface of the resin composite, the beams diffuse through the filler particles, which interact with the light and lead it to scatter in different directions ^5^. This phenomenon can be called structural, and factors including restoration size/depth, material translucency, and background color could influence the color-matching effect ^1^.

Previous laboratory studies comparing the performance of single-shade resin composites at different restoration depths found controversial findings. In two of these studies ^6^ ^,^ ^7^, unacceptable color matching was found for a single-shade resin (i.e., Omnichroma, Tokuyama, Tokyo, Japan) at different depths ^6^. Other studies, one using artificial acrylic teeth (i.e., A2 and A4 shades) at 1.5- and 3-mm restoration depths and another using circumferential disks made with multi-shade resin composite (Estelite Omega, Tokuyama, Tokyo, Japan, A3B shade) at 1.2- and 3-mm restoration depths also have not found satisfactory color matching. Meanwhile, a study assessing class V cavities prepared in acrylic teeth and filled with Omnichroma (i.e., A3 shade) found a better color matching at 3 mm than at 2 mm depth ^8^. This finding contradicts data that mention that, generally, the smaller the cavity, the better the color matching of the material and the smaller the color difference is (ΔE) ^9^.

Considering there is still limited research and information regarding the color matching of single-shade resin composites, this in vitro study aimed to evaluate the influence of two restoration depths and translucency on the color matching of single-shade resin composites compared with a multi-shade material through visual and instrumental analysis (with CIEDE2000 calculations, obtaining ΔE_00_ values). The tested hypothesis is that restoration depth (i.e., 2-and-4mm) could influence the color matching of single-shade resin composites. Additionally, the hypothesis that the translucency of these resins could be affected by the thickness was tested.

## Materials and methods

This study was reported according to CRIS Guidelines (Checklist for Reporting In Vitro Studies). The methodology was performed according to adaptations of previous studies ^10^.

### Sample selection

Specimens were prepared using artificial acrylic molar teeth (Pop Dent, DentBras, São Paulo, Brazil, Model 32M) 62 shade, which corresponded to the shades A2 and A3. These specimens were previously evaluated in a pilot test using a Vita Easyshade 4.0 spectrophotometer (Vita Zahnfabrik, Bad Sackingen, Germany). The shade was chosen once the A2 and A3 shades were among the most frequent Vita shades found ^10^. For this purpose, class I circular cavities were prepared in the center of artificial acrylic molars teeth on the occlusal surface with the aid of a PM21 diamond bur (American Burrs, Palhoça, SC, Brazil), in low rotation, with a diameter of 6 mm and with two different depths in relation to the cavo-surface angle (2-and-4 mm).

**Figure 1:**
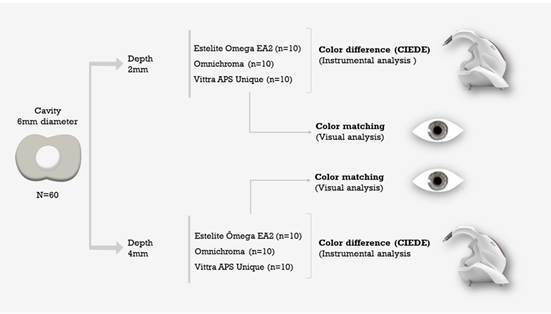
Study design with experimental groups at two different depths (2 and 4 mm), and resin composites evaluated through instrumental and visual analysis.

Figure 1 summarizes the study design. To fabricate the restorations, Single Bond Universal adhesive (3M, Saint Paul, Minnesota, EUA) was applied in the self-etch mode according to the manufacturer's instructions. The adhesive was actively applied with a disposable micro applicator for approximately 20 seconds, and a light jet of air was used over the liquid for approximately 5 seconds. Then, light curing was performed for 10 seconds with the Valo Grand Cordless curing light (Ultradent, USA) multiwave curing unit (1400 mW/cm² - measured with the radiometer Marc^®^ - BlueLight Analytics). Then, the teeth were separated into two groups according to the preparation depth: 2- and 4-mm.

For the translucency analysis, discs were prepared with each test resin composite, with 6 mm in diameter and thicknesses of 2-and-4 mm. For this, elastomeric matrices were used, where the material was accommodated and pressed with polyester strips.

According to previous studies ^3^, a convenience sample of 10 teeth per depth was used. Within each depth, the following single-shade resin composites were applied (n=10): Omnichroma (OC; Tokuyama, Tokyo, Japan) and Vittra APS Unique (VU; FGM, Joinville, SC, Brazil). The multi-shade resin composite Estelite Omega (EO; Tokuyama, Tokyo, Japan) in the EA2 shade was used as a control based on a previous study ^11^. Shade EA2 was chosen to be the control based on literature data, due to the translucency level of tested single-shade resin composites that would be more similar to an enamel shade.

Each resin was used according to the manufacturer's instructions, and light-curing was performed with a Valo Grand Cordless multiwave curing light (Ultradent, USA). The time indicated for the light-activation of each material is shown in Table 1. The restorations were stored in distilled water and then polished with rubber tips (Ultra Gloss, American Burrs, Palhoça, SC, Brazil) at low speed.

**Table 1:** Characteristics from de resin composites evaluated in the study

Abbreviation/Resin/ Lot	Shade	Filler content	Filler type	Matrix	Application
EO: Estelite Omega (Tokuyama), Lot 180E01	A2 enamel	Spherical zirconia-silica o (100-300 nm; mean: 200 nm)	Supra-nanometric	Bis-GMA, TEGDMA	Applied in 2mm increments and photoactivated by four seconds*
OC: Omnichroma (Tokuyama), Lot 040EY1	Universal	260 nm spherical (SiO_2_- ZrO_2_)	Supra-nano spherical	UDMA, TEGDMA	Applied in 2mm increments and photoactivated by six seconds*
VI: Vittra APS Unique (FGM), Lot 230921	Universal	Nano spheres of a complex of silica (200 nm) - zirconia	Nanohybrid	UDMA, TEGDMA	Applied in 2mm increments and photoactivated by eight seconds*

*The curing light used was activated in high power mode, with 1400 mW/cm^2^.

### Instrumental analysis of color matching

The definition of instrumental analysis involves using devices such as spectrophotometers, colorimeters, spectroradiometers, or digital cameras to help obtain color differences ^3^ ^,^ ^4^. The instrumental analysis to evaluate color matching was performed using a Vita Easyshade 4.0 spectrophotometer (Vita Zahnfabrik, Bad Sackingen, Germany), calibrated immediately before each series of measurements in a visualization booth with D65 lighting, which corresponds to average daylight. For readings of the CIEL *a*b* color coordinates, the samples were positioned on a neutral gray background and the tip of the spectrophotometer was positioned at the central point of the restoration and a point on the vestibular surface of the tooth, in the occlusal middle, according to a previous study ^12^. Readings were taken in triplicate, and an average of the L*, a*, and b* data was obtained for each specimen. Among the color parameters, L* refers to the brightness coordinate with a value ranging from zero (black) to 100 (white). The a* and b* values are the chroma coordinates on the red-green and yellow-blue color axes, respectively. Positive a* values indicate a shift to red and negative values indicate a change to green. Likewise, positive b* values indicate the yellow color range and negative values indicate the blue color range.

The calculation of the color difference (ΔE_00_) was obtained through the averages of the readings. The following CIEDE2000 formula was applied for each reading in the Microsoft Office Excel software (Microsoft Corporation, Redmond, WA, USA).

$$∆E_{00}={\left[{\left(\frac{∆L´}{K_{L}S_{L}}\right)}^{2}+{\left(\frac{∆C´}{K_{C}S_{C}}\right)}^{2}+{\left(\frac{∆H´}{K_{H}S_{H}}\right)}^{2}+\;R_{T\;}\left(\frac{∆C´}{K_{C}S_{C}}\right)\left(\frac{∆H´}{K_{H}S_{H}}\right)\right]}^{\frac{1}{2}}$$

Where:

ΔE_00_ = color difference for two points;

ΔL’ = light difference for two points;

ΔC’ = chroma difference to two points;

ΔH’= hue difference for two points;

SL; SC, SH = weighting functions (for adjusting the total color difference for variation in the location of the color difference pair in coordinates L', a', b.'

kL, kC, kH = parametric factors = 1 (correction terms for experimental conditions);

RT= rotation function (responsible for the interaction between chroma and hue differences in the blue region).

Table 2 shows the ΔE_00_ threshold values and their interpretation according to a previous study.

**Table 2:** Scores of color difference and color matching and their interpretation

Color difference (ΔE_00_) acceptability threshold and interpretation for color adjustment	
ΔE_00_ Threshold	Interpretation
≤ 0.8	Excellent match
> 0.8 ≤ 1.8	Acceptable match
> 1.8 ≤ 3.6	Mismatch (moderately unacceptable)
> 3.6 ≤ 5.4	Mismatch (clearly unacceptable)
> 5.4	Mismatch (extremely unacceptable)
Visual scale with color matching correspondence	
Visual Scale	Color matching
0	Excellent match
1	Very good match
2	Not so good
3	Obvious mismatch
4	Huge mismatch

### Visual analysis

Three independent and blinded evaluators performed a visual analysis to evaluate color matching. All specimens were randomly distributed at Microsoft Excel software (Microsoft Corporation, Redmond, WA, USA). Each tooth was numerically identified in its lower portion so that no evaluators could identify it. To carry out the measurement, the samples were arranged in a viewing booth on a neutral gray background, under D65 lighting, with an angle of approximately 45 degrees and about 30 cm from the viewer. To avoid visual fatigue, observers looked at a blue rubber dam at each color assessment ^10^. Subsequently, the evaluators with no difficulty in chromatic distinctions and with an experienced eye on dental restorations (all with post-graduation in operative dentistry) rated the color matching of each specimen on a scale from zero (0) to four ^4^ according to the correspondence of color between tooth and restoration as described in Table 2 ^10^. Each evaluator read all specimens twice, randomly and blinded to the material used. The reading value for each tooth corresponded to the average of the six readings (three raters with two readings per specimen).

### Translucency

The translucency of the three resin composites was evaluated using the Vita Easyshade 4.0 spectrophotometer (Vita Zahnfabrik, Bad Sackingen, Germany) to measure the L*, a*, and b* coordinates of each specimen on standardized black and white backgrounds. The spectrophotometer was calibrated immediately before each series of measurements and all the evaluations were performed with D65 lighting, which corresponds to average daylight. Then, the values obtained in each background were used to obtain the ΔE_00_ value through the CIEDE2000 formula and, consequently, determine the translucency level of each specimen. The results for each specimen were applied in the Microsoft Office Excel software (Microsoft Corporation, Redmond, WA, USA).

$$∆E_{00}={\left[{\left(\frac{∆L´}{K_{L}S_{L}}\right)}^{2}+{\left(\frac{∆C´}{K_{C}S_{C}}\right)}^{2}+{\left(\frac{∆H´}{K_{H}S_{H}}\right)}^{2}+\;R_{T\;}\left(\frac{∆C´}{K_{C}S_{C}}\right)\left(\frac{∆H´}{K_{H}S_{H}}\right)\right]}^{\frac{1}{2}}$$

Where:

ΔE_00_ = color difference for black and white backgrounds;

ΔL’ = light difference for black and white backgrounds;

ΔC’ = chroma difference to black and white backgrounds;

ΔH’= hue difference for black and white backgrounds;

SL; SC, SH = weighting functions (for adjusting the total color difference for variation in the location of the color difference pair in coordinates L', a', b.'

kL, kC, kH = parametric factors = 1 (correction terms for experimental conditions);

RT= rotation function (responsible for the interaction between chroma and hue differences in the blue region).

### Statistical analysis

The data were assessed for normality and homogeneity of variance using Shapiro-Wilk and Levene's tests. Instrumental analysis data were analyzed with a Two-Way ANOVA, considering factors of material and depth, followed by a post-hoc Tukey’s test. Visual analysis comparisons were conducted using the Kruskal-Wallis test, also followed by a post-hoc Tukey’s test. Translucency analysis data were evaluated using the Friedman test, with a subsequent post-hoc Tukey’s test. The study considered resin composites and restoration depths as factors, maintaining a significance level of 5%. Statistical analyses were performed using SigmaPlot 12.0 (Systat Software Inc., Chicago, USA).

## Results

### Instrumental analysis

The Two-Way ANOVA revealed significant main effects for both the factor group (p<0.001) and factor depth (p<0.001), as well as a significant interaction between group and depth (p=0.031). According to the instrumental analysis (Table 3), the restoration depth influenced the ΔE_00_ values of the Omnichroma and Vittra Unique single-shade resin composites (OC: p=0.012; VU: p<0.001). However, it did not significantly affect the ΔE_00_ in the control with a multi-shade resin (EO: p=0.273). Comparing the 2-and-4 mm depths, the color difference (ΔE_00_) was statistically higher in the 4 mm depth, with a higher ΔE_00_ for both single-shade materials (p<0.001).

**Table 3:** Mean results of color difference (ΔE_00_) values (±standard deviation) and classification of match results of instrumental analysis

Resin composites	Depth		Classification of match results	
	2 mm	4 mm	2 mm	4mm
OC	3.5 (1.1) ^Aa^	4.7 (0.9) ^Ab^	Moderately unacceptable	Clearly unacceptable
VU	6.9 (0.7) ^Ba^	9.1 (1.2) ^Bb^	Extremely unacceptable	Extremely unacceptable
EO	3.8 (0.8) ^Aa^	4.3 (1.1) ^Aa^	Moderately unacceptable	Clearly unacceptable

Different capital letters indicate statistically significant differences in the same column (p<0.05). Different lowercase letters indicate statistically significant differences in the same line (p<0.05).

Considering the same depth, the color difference (ΔE_00_) of the Vittra Unique single-shade resin was statistically significantly higher than Omnichroma and Estelite Omega for 2 mm and 4 mm (p<0.001). On the other hand, there was no statistically significant difference between Omnichroma and Estelite in each depth (2mm: p=0.886; 4mm: p=0.565).

Even though the materials present differences in their ΔE_00_ values, all groups presented unacceptable values of color differences concerning the CIEDE2000 reference values since the acceptability limit is 1.8 ^13^. Considering the color difference acceptability thresholds to single-shade materials, only the restorations with 2 mm Omnichroma presented a moderately unacceptable mismatch. In contrast, the restorations with the same material but with a depth of 4 mm presented a clearly unacceptable mismatch, as well as the restorations with the multi-shade material at 4 mm depth. The Vittra Unique restorations showed an extremely unacceptable mismatch in both depths.

### Visual analysis

**Figure 2:**
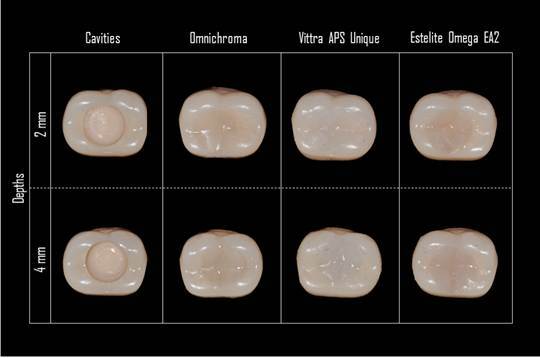
Study specimens from each group at different depths (2 and 4 mm).

The Two-Way ANOVA revealed significant main effects for the factor group (p<0.001), but non-significant effects for the factor depth (p=0.700) and the interaction between group and depth (p=0.451). Figure 2 shows the cavitated and restored specimens. In the visual analysis (Table 4), the depth did not influence color matching and no statistically significant differences were found for the same resin in different depths (p=0.451). Considering materials at the same depth, Vittra Unique had the lower color-matching values, followed by Estelite Omega and Omnichroma (p<0.001).

**Table 4:** Color matching mean values (**±**standard deviation) measured and classification of match results by visual analysis.

Resin composites	Depth		Classification of match results	
	2 mm	4 mm	2 mm	4 mm
OC	2.4 (0.3)^Aa^	2.4 (0.3)^Aa^	Not so good	Not so good
VU	0.2 (0.2)^Ba^	0.5 (0.4)^Ba^	Excellent match	Very good match
EO	2.0 (0.9)^Ca^	1.9 (0.5)^Ca^	Not so good	Not so good

Different capital letters indicate statistically significant differences in the same column (p<0.05). Different lowercase letters indicate statistically significant differences in the same line (p<0.05).

At both depths, Omnichroma had the highest score values (2.42), representing a higher color difference and smaller color matching, classified as “not so good” (level 2 of the scale). Meanwhile, the Estelite Omega multi-shade material was evaluated at intermediate values of color matching (1.9 and 2) and was categorized as a “not so good” match. Finally, the Vittra Unique resin showed the lowest score values (0.3 and 0.5), showing a lower color difference and ranging from an “excellent match” (level 0 of the scale) to a “very good match” (level 1 of the scale).

### Translucency

The Two-Way ANOVA revealed significant main effects for both the factor group (p<0.001) and factor depth (p<0.001), as well as a significant interaction between group and depth (p<0.001). Table 5 states the mean values of translucency for each group. Both single-shade resin composites showed a higher translucency than conventional resin at 2-mm (OC vs. EO: p<0.001; VU vs. EO: p<0.001) and 4-mm depth (OC vs. EO: p=0.002; VU vs. EO: p=0.001). The highest translucency values were found for the 2mm specimens, with OC being the most translucent resin composite, followed by VU and EO, with a statistically significant difference between all the groups (p<0.001). On the other hand, at 4 mm thickness, OC and VU did not show a statistically significant difference between them (p=0.983), with higher levels of translucency compared to EO (OC vs. EO: p=0,002; VU vs. EO: p=0,001). In all groups, translucency decreased with increasing specimen thickness.

**Table 5:** Translucency mean values (±standard deviation) measured by color difference (ΔE_00_) values (black and white background)

Resin composites	Depth	
	2 mm	4 mm
OC	15.3 (2.2) ^Aa^	4.3 (2.4) ^Ab^
VU	10.0 (1.1) ^Ba^	4.4 (1.4) ^Ab^
EO	7.1 (1.0) ^Ca^	1.6 (0.6) ^Bb^

Different capital letters indicate statistically significant differences in the same column (p< <0.05). Different lowercase letters indicate statistically significant differences in the same line (p<0.05).

## Discussion

Single-shade resin composites were developed to facilitate clinical practice, as they do not require the color selection step inherent in conventional multi-shade resin composites ^14^. Different manufacturers have developed many single-shade resin composites in recent years, and inconsistencies in the performance have been reported, especially about optical properties ^10^ ^,^ ^15^ ^,^ ^16^. Considering the results of this study, the hypothesis. Were accepted since the color matching decreased with the increase in depth (instrumental analysis) and the translucency was affected by the thickness of the resin composite.

The values measured in the instrumental analysis suggest that the restoration's depth influenced the color matching of the single-shade resin composites tested in this study. This data aligns with previous studies ^17^ ^,^ ^18^, in which the color difference increased in smaller cavities and the color matching was smaller. In this sense, the increased restoration depth could decrease the resin translucency ^15^. Considering that color matching is directly influenced by translucency ^10^, an increase in the material thickness may decrease its translucency ^17^. On the other hand, other studies that evaluated cavities with depths that ranged from 1 to 3 mm have not found differences between single-shade resin composites ^6^, probably due to the smaller depths evaluated than our study (i.e., up to 4mm). A previous study that evaluated single-shade resin composites at greater depth (i.e., 5 mm) also found a color matching of an unacceptable mismatch for Omnichroma applied in Class V cavities of human teeth extracted ^19^. In the present study, the ΔE_00_ values of both single-shade resin composites tested Omnichroma and Vittra Unique were considered with unacceptable mismatch according to the CIEDE2000 threshold of acceptability of 1.8 ^13^. Considering the Omnichroma resin, other studies have also found similar results ^10^ ^,^ ^20^. Although the ΔE_00_ values for the tested resin composites were high, the Omnichroma had a similar color difference to the Estelite Omega control. Therefore, the single-shade resin behaved similarly to the multi-shade resin without the need to select the color of the conventional resin composite.

The different depths evaluated could also have influenced the results found in this study. This finding is in agreement with the results of a previous study ^21^ but contradicts other studies where the depth did not influence the color matching ^6^ ^,^ ^7^. In restorations of only 2 mm, a previous laboratory study with Vittra Unique and Omnichroma found acceptable color matching in extracted human teeth ^3^. Besides, the variability in results can be attributed to the difference in methodologies used for color evaluation. In this study ^3^, the authors evaluated color matching through a calculation of the color adjustment potential (CAP), which takes into account the interaction between physical and perceptual components of the color. On the other hand, considering the ΔE_00_ values for the resin composites without applying it to the CAP calculation, the resin composites would present an unacceptable mismatch, as in our study, since the ΔE_00_ values corresponded to 5.43 and 4.75 for Omnichroma and Vittra Unique, respectively, with values higher than the acceptability limit of 1.8.

The high values of ΔE_00_ found in our study could also be associated with the type of specimen used (acrylic teeth), which differs from a natural tooth. In other studies, the specimens were not only acrylic teeth with class III and class I preparations ^10^, but also human teeth extracted ^3^ ^,^ ^22^, as well as a clinical trial in anterior teeth with class V and III ^20^. Natural teeth are polychromatic, curved, translucent, and multi-layered, altering the light reflected on them and, consequently, on restorative materials. However, standardization of human teeth color to be used as specimens in a laboratory study and the ethical issues involved are not accessible. Although our study used specimens of acrylic teeth, these specimens were mainly used in previous laboratory studies ^10^, which facilitates comparability between them. Other studies with natural teeth found similar results to those with artificial specimens, extracted teeth ^1^, or even teeth in a clinical trial.

The analysis of the color matching and difference has been evaluated in vitro respectively through visual tests and instrumental analyses using devices such as spectrophotometers, colorimeters, spectroradiometers, or digital cameras ^3^ ^,^ ^4^. The intraoral spectrophotometer used in our instrumental analysis (VITA Easyshade; VITA Zahnfabrik, Bad Säckingen, Germany) is widely used in color research in dentistry because it is a suitable, convenient, and feasible method, which facilitates reproducibility for comparison with other studies. Different ways of color difference analysis (ΔE) have been proposed in the literature ^10^, and the CIEDE2000 formula represents better the parameters of perceptibility and acceptability of color by human eyes than the CIELAB formula. Due to this, the CIEDE2000 was chosen for our experiment.

Both visual and instrumental methods are valid and recommended to evaluate optical properties in dentistry. According to the FDI resin composite evaluation criteria, the visual evaluation method must be chosen when considering color matching. Visual analysis is the most frequently used method and is fundamental for evaluating the color of restorations since color depends on optical illusion phenomena, which are not measurable by devices ^23^. Furthermore, the guide indicates using instrumental methods, such as spectrophotometers and colorimeters, due to their satisfactory accuracy and reliability.

In our study, the instrumental and visual analysis results differed. In contrast, another study ^3^ found similar results for both methods used to evaluate Omnichroma and Vittra Unique resin composites in extracted human incisors with a circular cavity at 2 mm depth. However, another previous study found a high color difference to Omnichroma (greater than the acceptability threshold) and unacceptable color matching, but visually the restoration was considered reasonable probably due to the high material's translucency. The mechanisms involved in the color of resin composites still need to be fully understood. Due to the high complexity of the human visual system, there is no single explanation of how color-related visual phenomena occur and this complexity may be related to this disparity between the results.

The human nervous system seeks to logically interpret what the eyes see, often causing optical illusion phenomena, where the brain processes an image different from what the eyes see ^4^. This optical illusion phenomenon is a psycho-physical phenomenon, which is “visually perceptible, but not quantifiable or measurable by any instrument” and “is not modeled by the current CIELAB mathematical model” according to The International Organization for Standardization (ISO) technical report no. 28642. These phenomena could explain the differences between the instrumental and visual analysis results. In this context, it is reaffirmed, the importance of analysis by subjective methods and not only objective methods, since the color of the resin composite will be classified in the clinical context as aesthetically pleasing or not, according to the perception of the human eye, by the patient and the professional.

The level of aesthetic demand of the professional and the patient can differ. Due to this, the visual analysis is subject to variability between the criteria used by each examiner, which can result in subjective evaluations that make comparability difficult, unlike the instrumental analysis ^24^. One study ^20^ that evaluated color difference in restorations (class V and III), in patients, performed with Omnichroma (Tokuyama, Japan), visually analyzed by two Ph.D students, showed an unacceptable color matching to them, but high acceptability among patients ^20^. Some studies have reported that tooth shape, position, and color are the most important factors for patient satisfaction ^24^. The patient satisfaction criteria are also an essential factor to be considered according to the FDI.

One variable that could influence color matching and that was evaluated in this study is translucency, an optical property of dental tissues and materials related to the ability to transmit light ^9^. Thus, translucency may contribute to material color matching by allowing adjacent substrate features to shine through ^25^. Translucency seems to be a desirable property in single-shade resin composites since the Omnichroma manufacturer indicates that the material undergoes translucency optimization after light activation, which aims to facilitate color matching. In this scenario, since the material tends to present greater translucency, the type of control resin composite used was enamel, which is more translucent than dentin. Some previous similar studies used enamel resin composites to compare with single-shade resin composites ^9^ ^,^ ^16^ ^,^ ^23^. A previous laboratory study with the same single-shade resin composites from this study (OC and VU) corroborates this statement and also showed that the VU resin composite also increases its translucency after polymerization ^26^. Also, the manufacturers of OC and VU recommend applying an underlying layer of opaque material when the substrate is dark or aesthetically undesirable. This suggests that single-shade resin composites mimic the color by visualizing the underlying substrate due to their translucency ^18^.

The single-shade resin composites work by structural color, being dependent on the interaction of light through the material, where the light interacts with nanostructures, occurring diffraction, interference, or scattering phenomena (different from conventional resin composites, which have pigments that absorb and reflect light generating colors) ^6^. In this study, we found higher translucency scores for single-shade materials in relation to multishade resin composite, at both depths and the translucency decreased with an increase of the specimen’s thickness.

The material's translucency is directly linked to its value or amount of white and black. The latter is the most important of hue, and chroma color properties, as the human eye is more sensitive to light/dark changes than hue and chroma ^10^. The best results found in our visual analysis were for Vittra Unique resin composite, ranging from an “excellent match” to “very good match. On the other hand, Omnichroma and Estelite Omega presented results of "not so good." The OC and VU may have a difference in value level since OC had a darker appearance (lower value) in relation to restorations performed with VU, which had a lighter appearance (higher value). This finding corroborates the results of a previous study that showed that restorations made with VU have a higher whitening index for dentistry (ΔW_ID_) than those made with OC ^27^. Colors with higher values tend to generate more harmonic aspects to the human eye, which probably explains the better color-matching scores obtained to VU in the visual analysis. In this way, a greater color difference value (ΔE_00_) cannot always mean an esthetically unfavorable restoration, since this greater color difference may be related to tooth whitening. When comparing the different single-shade brands, OC showed greater translucency at 2 mm and similar results to VU at 4 mm (p<0.05). Some variables can influence the translucency parameter of the material, such as shade, thickness, matrix composition, and filler particle (size and content). An increase in the filler content of resin composite is associated with increased material opacity and a consequent reduction in translucency. Vittra Unique and Ominichroma resin composites have a slight difference in their fillers. While Vittra Unique has 82% by weight and 72% by volume of nanohybrid fillers, Ominichroma has 79% by weight and 68% by volume of supra nanometric fillers ^3^. Both materials showed differences in color matching in the visual analysis. However, since the translucency parameters are influenced by other variables, probably, other composition factors explain the differences between the translucency parameters of OC, VU, and EO. 

Regarding material composition, the organization of the material structure may influence its optical properties by causing different interactions with light beams ^3^. These structures selectively reflect and absorb specific wavelengths for specific colors ^4^. When the light illuminates the material, the fillers of charges scatter the light beams in different directions ^3^. Spherical symmetric nanoparticles with a diameter of 200 to 300 nm (smaller than the wavelength of visible light) could produce the phenomenon of structural color, where there is no addition of pigments. Examples of these particles are the silica found in the single-shade resin composites evaluated (Omnichroma and Vittra Unique) ^5^. Besides, Omnichroma and Vittra Unique have the same organic matrix (Urethane Dimethacrylate (UDMA) and Triethylene glycol dimethacrylate (TEGDMA)), and both have silica and zirconia fillers but with different size and distribution. The Vittra Unique presents nanohybrid fillers with a diameter of 200 nm of silica. On the other hand, Ominichroma presents spherical and uniform supra nanometric charges of 260 nm of silica and zirconia. The manufacturer of Omnichroma (Tokuyama Dental, Tokyo, Japan) states that the chameleon effect of its resin is due to its spherical and uniform charges, with a diameter of 260 nm, which are responsible for generating the reflection of waves with wavelengths in order from red to yellow, making it easier to match natural tooth hues ^14^. Meanwhile, the manufacturer of Vittra Unique (FGM, Joinville, SC, Brazil) does not clearly explain the mechanisms involved in color matching, only citing the increase in translucency after light-activation ^21^. These differences in the composition may also be responsible for different results of color difference and matching found, respectively, in the instrumental and visual analyses between the materials.

Regarding the control used, the multi-shade resin composite Estelite Omega has an organic matrix with Bis-GMA (bisphenol A-glycidyl methacrylate) and TEGDMA (triethylene glycol dimethacrylate). The color matching of this nanofilled material was previously tested. The authors found the material could not be used to restore any tooth color due to its limited chameleon effect. The multi-shade resin composite has a low potential for color matching through light reflection from the background dentin. Therefore, to obtain an acceptable color matching with this type of material, a specific combination of different shades of resin composites would be required ^17^. These findings agree with those found in the present study. In addition, the absence of layering with resin composite in the shade “dentin” combined with “enamel” may have contributed to the high ΔE values since the simulation of natural optical properties must be performed with the combination of layers of resin composites with different levels of opacity ^10^. It was only used the Estelite Omega of enamel shade in our study to better mimic a single-shade resin with a higher translucency. Due to this, multi-shade resin composite of enamel shade is mainly used as control of studies that aim to evaluate the color matching of single-shade materials ^9^ ^,^ ^10^.

The findings of this study are essential to fill the existing gap in the literature about single-shade resin composites. A limitation of laboratory studies is that they may need to simulate clinical conditions adequately. Restorations are perceived differently in the oral and laboratory environments. Factors such as the dental shape and location and the interaction of teeth with soft tissues are only present in the clinical setting and alter the perception of color. Besides, the optical properties are material dependent ^21^, and inferences from the single-shade resin composites evaluated by this study may not be extrapolated to other single-shade resin composites. A previous study showed that in addition to restoration thickness, other factors such as background and surrounding shade can affect the mimicry ability of single-shade resin composites ^28^. Therefore, single-shade resin composites may have their color-matching ability varying according to the scenario in which they are used. Manufacturers do not indicate a maximum/ideal amount that the material can be used without compromising color matching and translucency properties, which may be a limitation to its use. Manufacturers only recommend that an opaque support resin composite be used when there is no back wall to reproduce the color (e.g. class III and IV) in order to block the influence of colors from other parts of the mouth and/or if the dental substrate is darkened.

Finally, it is essential to consider that the reasons for the failure of direct restorations in the long term are different between anterior and posterior teeth. In a previous clinical study with direct restorations evaluated after 20 years, it was found that esthetic failures are predominant in anterior teeth, while fractures are the leading cause of failure of posterior restorations. Therefore, the aesthetics of posterior teeth would be less critical than anterior teeth and mechanical strength would be the most crucial property. Considering the demands required by each group of teeth, all materials evaluated in this study present reasonable aesthetics and could be applied in the posterior region but probably would not be indicated for anterior teeth. This unfavorable esthetic performance of Omnichroma on anterior teeth has already been found in a clinical study ^20^. Despite the ΔE results corresponding to values above the acceptability limit, using single-shade resin composites seems advantageous for the posterior region since this region does not have a high aesthetic demand. Besides, this type of resin composite could reduce chair time, since it does not require the color selection step, and provide financial savings by reducing the required color inventory of multi-shade resin composites. In this context, this material can be also a good input option for the public service, where there is often a shortage of resources. Further studies are indicated for more detailed investigations of different single-shade resin composites.

The instrumental and translucency analysis results suggest that color matching may vary with increasing depth of the single-shade resin. Besides, the single-shade resin composite Ominichroma had a similar color matching to a multi-shade resin at 2 and 4-mm depth. On the other hand, in the visual analysis the depth did not affect color matching and Vittra Unique had the best matching results.

## Conclusion

The instrumental and translucency analysis results suggest that color matching may vary with increasing depth of the single-shade resin. Besides, the single-shade resin composite Ominichroma had a similar color matching to a multi-shade resin at 2 and 4-mm depth. On the other hand, in the visual analysis the depth did not affect color matching and Vittra Unique had the best matching results.
